# Flor Yeast: New Perspectives Beyond Wine Aging

**DOI:** 10.3389/fmicb.2016.00503

**Published:** 2016-04-14

**Authors:** Jean-Luc Legras, Jaime Moreno-Garcia, Severino Zara, Giacomo Zara, Teresa Garcia-Martinez, Juan C. Mauricio, Ilaria Mannazzu, Anna L. Coi, Marc Bou Zeidan, Sylvie Dequin, Juan Moreno, Marilena Budroni

**Affiliations:** ^1^SPO, Institut National de la Recherche Agronomique – SupAgro, Université de MontpellierMontpellier, France; ^2^Department of Microbiology, Agrifood Campus of International Excellence ceiA3, University of CordobaCordoba, Spain; ^3^Department of Agricultural Sciences, University of SassariSassari, Italy; ^4^Department of Agri-Food Sciences, Holy Spirit University of KaslikJounieh, Lebanon; ^5^Department of Agricultural Chemistry, Agrifood Campus of International Excellence ceiA3, University of CordobaCordoba, Spain

**Keywords:** flor yeast, wine, biofilm, -omic tools, immobilization, biofilm management, biocapsules

## Abstract

The most important dogma in white-wine production is the preservation of the wine aroma and the limitation of the oxidative action of oxygen. In contrast, the aging of Sherry and Sherry-like wines is an aerobic process that depends on the oxidative activity of flor strains of *Saccharomyces cerevisiae*. Under depletion of nitrogen and fermentable carbon sources, these yeast produce aggregates of floating cells and form an air–liquid biofilm on the wine surface, which is also known as velum or flor. This behavior is due to genetic and metabolic peculiarities that differentiate flor yeast from other wine yeast. This review will focus first on the most updated data obtained through the analysis of flor yeast with -*omic* tools. Comparative genomics, proteomics, and metabolomics of flor and wine yeast strains are shedding new light on several features of these special yeast, and in particular, they have revealed the extent of proteome remodeling imposed by the biofilm life-style. Finally, new insights in terms of promotion and inhibition of biofilm formation through small molecules, amino acids, and di/tri-peptides, and novel possibilities for the exploitation of biofilm immobilization within a fungal hyphae framework, will be discussed.

## Introduction

*Saccharomyces cerevisiae* flor yeast are responsible for the biological aging of Sherry and Sherry-like wines. The main feature of these yeast is that at the end of alcoholic fermentation, when they are under nitrogen and sugar depletion, they shift from fermentative to oxidative metabolism (i.e., the diauxic shift) and rise to the wine surface to form multicellular aggregates. This aggregation leads to the build-up of a biofilm, or velum or flor (Esteve-Zarzoso et al., 2001; Aranda et al., 2002; Alexander, 2013).

Biofilm formation is strongly dependent on the nutritional status of the wine. It is well known that biofilm starts when the concentration of any fermentable carbon source is imperceptible or null (Martínez et al., 1997a). In addition, the presence of other carbon sources, such as glycerol and ethyl acetate, can induce biofilm formation (Zara et al., 2010). Thus, biofilm formation is not limited to aerobic growth on ethanol, but occurs also on other reduced non-fermentable carbon sources that provide sufficient energy input. Moreover, biofilm formation is affected by the availability of nitrogen. It has been shown that in wine lacking nitrogen sources, the flor yeast do not form a biofilm, and that the addition of amino acids to the medium does not induce biofilm formation (Mauricio et al., 2001; Berlanga et al., 2006). Zara et al. (2011) reported that biofilm formation is favored by addition of 37.5 mM ammonium sulfate, while when these concentrations exceed 150 mM, biofilm formation is prevented.

During biofilm growth, the lack of fermentable carbon sources and the availability of oxygen induce cells to maintain aerobic metabolism, which results in important changes to the wine sensorial and aromatic properties, and to its chemical composition. These changes include a reduction of the volatile acidity due to the metabolism of acetic acid, and production of acetaldehyde at the expense of ethanol. Moreover, acetaldehyde by-products provide the distinctive flavor of Sherry and Sherry-like wines, such as 1,1-diethoxyethane and sotolon (Dubois et al., 1976; Guichard et al., 1992; Moreno et al., 2005; Zea et al., 2015).

Oxidative metabolism is essential to allow flor strains to remain at the wine surface; indeed, Jiménez and Benítez (1988) demonstrated that flor *petite* mutants cannot form biofilm and are more sensitive to ethanol. Furthermore, sensitivity to ethanol is inversely correlated with rate of biofilm formation, where the less resistant strains produce the biofilm more rapidly (Martínez et al., 1997b).

**FIGURE 1:**
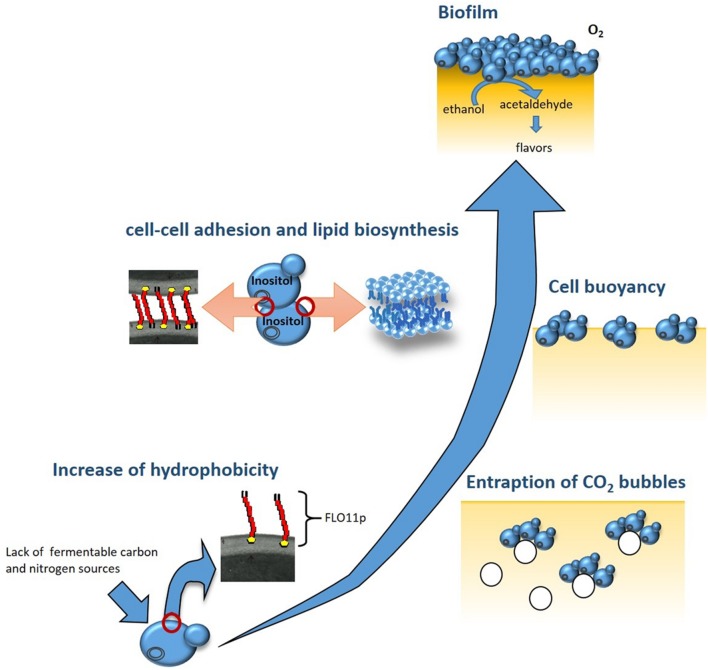
**Building of the biofilm by flor yeast.** At the end of fermentation, *Saccharomyces cerevisiae* flor yeast adapt to the lack of fermentable carbon and nitrogen sources by triggering specific metabolic pathways: cAMP-protein kinase A (PKA), mitogen-activated protein kinase (MAPK), and TOR. These, in turn, activate the transcription of *FLO11*, which codes for a hydrophobic protein. The higher cell surface hydrophobicity encourages formation of multicellularaggregates that entrap CO_2_ bubbles and float toward the wine surface. In biofilm cells overexpressing *FLO11*, inositol is used for the assembly of the glycosylphosphatidylinositol anchor of Flo11p. The decrease in the intracellular concentration of inositol and the availability of oxygen on the wine surface activate the expression of genes regulated by inositol–choline responsive elements, such as ACC1, which results in the *de-novo* biosynthesis of unsaturated fatty acids and in increased cell buoyancy. The different cell layers that constitute the mature biofilm protect the wine from direct exposure to oxygen, and induce important changes in the organoleptic, aromatic and chemical composition of the wine.

The ability of *S. cerevisiae* to adapt to environmental and nutritional changes depends on the activation of metabolic pathways that induce the expression of specific genes. For biofilm formation, expression of the *FLO11* gene has been shown to be the key event. Indeed, the increased expression of *FLO11* during the diauxic shift results in higher cell-surface hydrophobicity. This encourages the formation of multicellular aggregates that entrap CO_2_ bubbles deriving from the fermentation of the residual sugar, thus providing the buoyancy to the aggregates, and therefore promoting biofilm formation (Zara et al., 2005) (**Figure 1**). Activation of *FLO11* depends on three specific pathways: the cAMP-protein kinase A (PKA) pathway; the mitogen-activated protein kinase (MAPK) pathway; and the TOR pathway (Braus et al., 2003; Vinod et al., 2008). It has been shown that in biofilm-inducing media, biofilm formation and *FLO11* transcription can be significantly reduced by the addition of rapamycin, which is a well-known inhibitor of the TOR pathway, and the deletion of *RAS2*, which regulates the PKA and MAPK pathways (Zara et al., 2011). Finally, the expansion of minisatellites within the central domain of *FLO11* contributes to increased protein glycosylation and hydrophobicity of the Flo11 glycoprotein (Flo11p) of flor yeast (Reynolds and Fink, 2001; Zara et al., 2005; Fidalgo et al., 2006).

As well as the role of *FLO11* in the rising of cells and in their hydrophobicity, biofilm formation appears to be dependent on increased cell buoyancy. This is influenced by their lipid content and composition, as flor strains have greater chain lengths and unsaturation levels of their fatty-acid residues than those shown by non-biofilm-forming strains of *S. cerevisiae* (Farris et al., 1993; Zara et al., 2009). Addition of cerulenin, which is an antibiotic that inhibits *de-novo* fatty-acid biosynthesis, results in a dramatic reduction in *FLO11* transcription levels and biofilm weight of flor yeast grown in biofilm-inducing media (Zara et al., 2012). Inositol availability also affects biofilm formation, possibly due to its key role in the assembly of the glycosylphosphatidylinositol anchor of Flo11p, and in the regulation of lipid biosynthetic genes, such as *ACC1* (Zara et al., 2012).

Due to their metabolic and genetic peculiarities, flor strains can overcome stress caused by high ethanol and acetaldehyde contents in Sherry and Sherry-like wines (Budroni et al., 2005). It has been hypothesized that this adaptive ability is related to DNA mutations caused by acetaldehyde, such as double-strand breaks (Ristow et al., 1995). These mutations are considered to be responsible for mitochondrial DNA polymorphism (Castrejon et al., 2002) and for gross chromosomal rearrangements in flor yeast (Infante et al., 2003). The complexity and specificity of the flor yeast genome make these strains an interesting model for studies into speciation of *S. cerevisiae* and into adaptive evolution based on mutations in the *FLO11* gene (Fidalgo et al., 2006).

Considering that comparative genomics, proteomics and metabolomics of flor and wine yeast strains are shedding new light on several features of these special yeast, in this review we will discuss the most recent data obtained through the analysis of flor yeast with -*omic* tools. Moreover, we will report on new insights in terms of promotion and inhibition of biofilm formation through small molecules, amino acids and di/tri-peptides, and on novel possibilities for the exploitation of yeast immobilization within a fungal hyphae framework.

## Genetic Diversity Indicates That Most Flor Yeast Share the Same Origin

Biological aging is performed traditionally in several countries in Europe, including Hungary (Tokaj-Hegyalja) to produce Szamorodni, Italy (Sardinia) to produce Vernaccia di Oristano, Spain (Jerez area) to produce Xeres, and France (Jura) to produce Vin Jaune. Flor yeast isolated from the biofilms of these different wines have long been considered as specific varieties of *S. cerevisiae* given their unique behavior, although until recently we had no knowledge whether the strains in these different countries are related or not.

The first attempt to differentiate flor strains based on their ability to metabolize sugars (i.e., galactose, dextrose, lactose, maltose, melibiose, raffinose, sucrose) classified flor yeast into four varieties: *Saccharomyces cerevisiae var. beticus*, *Saccharomyces cerevisiae var. cheresiensis*, *Saccharomyces cerevisiae var. montuliensis* and *Saccharomyces cerevisiae var. rouxii* (Martínez et al., 1997b). Strains of these four varieties were also detected among Jura flor strains (Charpentier et al., 2009). More recent molecular studies have revealed that despite the high diversity detected for mitochondrial restriction fragment length polymorphism profiles, Spanish Sherry wine yeast share a specific 24-bp deletion in ITS1, which suggests a single family for Spanish Sherry yeast (Esteve-Zarzoso et al., 2001), while another allele of the ITS1 region has been detected among French flor strains (Charpentier et al., 2009). Given the different geographic origins and the genetic specificities of flor yeast, the question of the origin of flor yeast can be investigated further.

**FIGURE 2:**
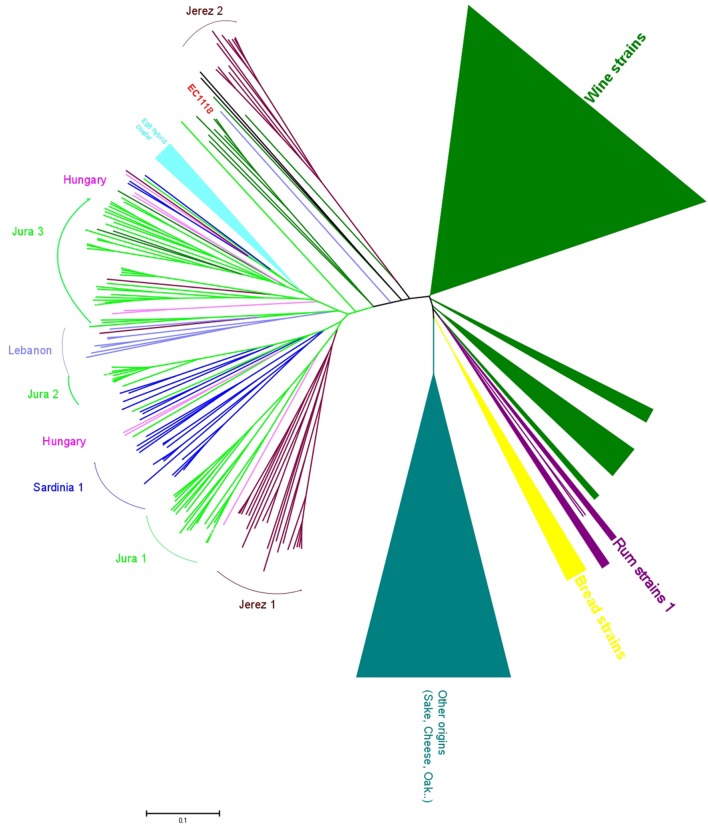
**Genomic diversity of flor strains.** Neighbor-joining tree built by evaluating the flor strains at 12 microsatellite loci, in comparison with strains of other origins. The tree was built from the Dc chord distance and drawn with MEGA5.22. Clusters of wine strains and other origins have been condensed due to their large size (modified from Legras et al., 2014).

The genetic analysis of flor strains with microsatellite typing revealed that, surprisingly, most flor strains of Spain, Italy, France and Hungary belong to the same genetic group of *S. cerevisiae* (Legras et al., 2014), with sub-clustering that corresponds to the strains from each of these countries (**Figure 2**). Of note, this sub-clustering might be related to differences in the ability to produce a biofilm. Some strains in the main group of Jura flor strains differ in terms of the length of the *FLO11* gene and the presence of a 111-bp deletion in *ICR1*, the long, non-coding RNA that regulates the expression of *FLO11*. Jura flor strains with this deletion produce thicker biofilms, whereas Jura strains with a longer *FLO11* allele and a wild-type version of *ICR1* produce thinner biofilms (Legras et al., 2014). Interestingly, two isolates from Hungary have a heterozygote version of the promoter: one wild-type and one that has the deletion. The presence of a single cluster of flor strains from different countries attests that they share a unique origin and indicates that flor yeast have migrated within Europe, as has been shown for wine yeast all over the world (Legras et al., 2007). In agreement with this hypothesis, some isolates related to flor strains have also been isolated in Lebanon. This recent characterisation of flor strains from different countries into a single group demonstrates the ecological success of these flor strains, which occupy the specific niche of the wine surface. This suggests that there are genomic specificities associated to the adaptation to the wine biological aging environment, such as has been seen for *FLO11*, and will be very likely for other genes.

## Adaptation of Flor Yeast and Copy-Number Variations

Comparative genome hybridization provided the first insight into the adaptation of wine (Dunn et al., 2005) and flor (Infante et al., 2003; Legras et al., 2014) yeast to their environment. Aneuploidies are frequently involved in adaptation to changing environments, as has been observed in adaptive evolution experiments (Dunham et al., 2002; Gresham et al., 2008). Sequences contained inside gross chromosomal rearrangements can be amplified, which leads to greater expression of some genes, and the first comparison of two flor strains was believed to indicate flor-strain peculiarities (Infante et al., 2003). However, the comparative genome hybridization profiles of six flor strains from Spain, Hungary, France and Italy, compared to those of wine strains and using S288C as a reference, did not reveal such complex aneuploidy profiles (Legras et al., 2014). This global comparison revealed a drop in the hybridisation signal in the sub-telomeric regions, which suggested missing or divergent genes in these regions. However, when looking for amplified genes, only three genes were detected: *FRE2*, *MCH2*, and *YKL222C* (Legras et al., 2014). *MCH2* is annotated as a putative monocarboxylic-acid transporter, although its involvement in monocarboxylic acid transport has not been shown experimentally (Makuc et al., 2001). *MCH2* is important for yeast survival during the second phase of alcoholic fermentation (i.e., alcohol accumulation) (Novo et al., 2013), and has been shown to be induced under vanillin stress and to confer vanillin resistance (Park et al., 2015). These results show that copy-number variations solely cannot explain the adaptation of flor yeast to their environment, as has been proposed previously (Infante et al., 2003). However, *MCH2* and *YKL222C* are promising targets, the roles of which need to be evaluated. Further studies, such as population genomics, have to be performed to unravel the genetics basis of flor-yeast adaptation.

## Specificities of Flor Yeast: Further Insights From Proteomics and Metabolomics

In recent years, proteomics and metabolomics have been applied to the study of flor yeast metabolism and their responses to environmental conditions. Moreno-García et al. (2014) performed a proteome analysis during biofilm formation that focussed on elucidation of the role of the mitochondria, which are the essential organelle for oxidative metabolism, for elaboration of several stress responses, and for the formation of biofilms (van Loon et al., 1986; Costa et al., 1993, 1997; Piper, 1999; Reinders et al., 2006; Ma and Liu, 2010; Fierro-Risco et al., 2013; Vandenbosch et al., 2013). From this proteome analysis, a number of mitochondrion-localized proteins that might be responsible for flor yeast behavior were highlighted. These included proteins involved in carbohydrate oxidative metabolism, biofilm formation, apoptosis, and responses to stresses typical of biological aging; e.g., ethanol, acetaldehyde, and reactive oxygen species (Fierro-Risco et al., 2013). Also, proteins associated with non-fermentable carbon uptake, glyoxylate and the TCA cycle, cellular respiration, and inositol metabolism are more expressed in yeast growing under biofilms than under fermentative conditions (Moreno-García et al., 2015b). Ino1p, which participates in inositol biosynthesis, was five-fold more expressed under biofilm conditions (Moreno-García et al., 2015b). Accordingly, Zara et al. (2012) reported that under biofilm-forming conditions, flor yeast show greater expression of genes involved in inositol biosynthesis.

The presence of proteins involved in cell-wall biosynthesis and protein glycosylation, which are important for cell-cell adhesion and hence for biofilm formation, has also been reported (Moreno-García et al., 2015b). Through the combination of proteomics and innovative metabolomics techniques that were aimed at quantifying minor volatile compounds under exhaustively controlled biofilm conditions, 33 proteins were shown to be directly involved in the metabolism of glycerol, ethanol and 17 aroma compounds (Moreno-García et al., 2015a). Although proteome analyses for oenological purposes have expanded substantially in recent years, particularly in terms of fermentative yeast (Zuzuarregui et al., 2006; Salvado et al., 2008; Rossignol et al., 2009), the relationships between changes in the yeast proteome and exometabolome and the influence of such changes on the organoleptic properties of wine still remained to be explored. The application of comparative -*omic* disciplines to flor yeast has provided novel knowledge on several features of these yeast, and has revealed the extent of the proteome remodeling that is imposed by the biofilm life-style. Under fermentative conditions, flor and wine yeast have comparable metabolism, although some differences have been revealed. For instance, unlike other fermentative strains, flor yeast increases the concentrations of some higher alcohols with their respective acetic acid esters and ethyl esters of C6 and C8 acids (Moreno et al., 1991). Furthermore, during the fermentation process and biofilm formation, flor strains yield higher levels of lactones than other non-flor strains (Zea et al., 1995). The intracellular accumulation and consequent excretion of terpenic compounds during fermentation, as well as during biofilm formation, was also shown by Zea et al. (1995).

Yeast growth under biofilm-forming conditions and wine biological aging are accompanied by the production of specific wine aromas. Systematic studies have shown that acetaldehyde is the most important metabolite in terms of the different levels between biologically aged and unaged wines, and have also highlighted the decrease in volatile acidity and glycerol content in aged wines (Mauricio et al., 1997, 2001; Cortés et al., 1998, 1999; Berlanga et al., 2004; Muñoz et al., 2005, 2007 and Moreno-García et al., 2013). Among the 35 aroma compounds quantified by Muñoz et al. (2005, 2007), acetaldehyde, 1,1-diethoxyethane, 2,3-butanediol (levo + meso forms), isoamyl alcohols, ethyl and isoamyl acetates, butanoic acid, 2,3-methylbutanoic acids, and 4-butyrolactone, were defined as the most active odorant compounds. Each of these showed odor activity values (OAVs) >0.8 in the biologically aged wines. Only 1-butanol, 2-butanol, isobutyl acetate, furanmethanol, and neral were present at levels 10-fold below their odor perception thresholds. The remaining compounds showed OAVs between 0.1 and 0.8. Compounds with OAVs >1 are considered as important contributors to the aroma of beverages, although there are exceptions when odorants with high OAVs are suppressed and compounds with lower OAVs are revealed as important contributors (Grosch, 2001). All of the 35 compounds studied here showed significant differences after biological aging, in relation to the initial control non-aerated wine (Cortés et al., 1998, 1999; Zea et al., 2001).

These data were confirmed and enriched by Muñoz et al. (2005, 2007) when they studied the effects of periodic aeration on metabolites such as acetaldehyde and its derivatives, and higher alcohols, their acetic-acid esters, and 3-(methylthio)-1-propanol, all of which increased in content as a consequence of the flor yeast growing under biofilm forming conditions. In contrast, the acids of 4, 5, and 6 carbon atoms showed lower concentrations in aged wines, and levels close to zero were obtained for 2-butanol, pantolactone, Z-whisky lactone, 4-ethylguaiacol, furanmethanol, 3-ethoxy-1-propanol and neral, after the same time of aging under biofilm forming conditions. Concentration changes obtained for other important aroma compounds, such as Z-whisky lactone and 4-ethylguaiacol, can only be explained because of the aging process carried out in contact with oak barrels.

The link between the intracellular proteins and metabolites excreted by yeast that are strongly related to sensorial properties constitutes a new and interesting advance in biological information systems. The knowledge generated can be considered as useful information for innovation in fermentative, winemaking and biotechnological-based industries in the near future.

## Flor Yeast as a Biological Model for the Study of Small Molecules That Inhibit or Promote Biofilm Formation

Microbial biofilms are tenacious structures that can be difficult to eradicate and to treat with the current arsenal of antifungal agents. This is mainly due to a lack of guidelines for biofilm management, and to difficulties in their diagnosis and identification. In contrast, many microbial biofilms are beneficial for a plethora of biotechnological processes, like cleaning up hazardous waste sites, filtering biofuels and wastewaters, and forming bio-barriers to protect soil and groundwater from contamination (Ashraf et al., 2014). Similarly for many food processes, such as maturation of cheese (Licitra et al., 2007) and biological aging of Sherry wines (Zara et al., 2005).

Problems related to biofilm eradication motivate current efforts to find compounds that can alter cell-surface hydrophobicity, typically through interactions with cell-wall components, and mainly the cell-wall mannoproteins, thus counteracting biofilm formation. Antimicrobial peptides are lead compounds in this approach. Many antimicrobial peptides have been shown to modulate adhesion and biofilm formation of some yeast and fungi due to hydrophobic and electrostatic interactions. For example, histidine-rich glycoproteins greatly inhibit biofilm formation by *Candida albicans* by binding and rupturing cell-wall components (Rydengard et al., 2008). In contrast, the antimicrobial peptides histatin-5 and LL-37 are antagonized by the cell-wall mucin Msb2 of *C. albicans*, which enhances resistance toward such compounds (Szafranski-Schneider et al., 2012).

In addition to antimicrobial peptides, other small molecules are currently being assessed for anti-biofilm activity. Zhao and Liu (2010) have shown that N-acetyl cysteine has anti-bacterial properties toward *Pseudomonas aeruginosa* and might mediate detachment of *P. aeruginosa* biofilms. A recent study reported that when mixed with other amino acids and nisin, L-cysteine prevents biofilm formation by *Streptococcus mutans* (Tong et al., 2014). Other studies on the effects of amino acids are controversial. Sanchez et al. (2013) reported that D-amino acids inhibit biofilm formation in *P. aeruginosa*, while Sarkar and Pires (2015) showed that they have no effect on *Bacillus subtilis*, *Staphylococcus aureus*, or *Staphylococcus epidermidis.* Moreover, a report of promising anti-biofilm activity of D-amino acids on *B. subtilis* strains (Kolodkin-Gal et al., 2010) was recently retracted (Hofer, 2014).

Nitrogen is a fundamental nutrient in living cells, and its metabolism is involved in major developmental decisions in *S. cerevisiae* (Forsberg and Ljungdahl, 2001). According to Homann et al. (2005), clinical and vineyard isolates of *S. cerevisiae* can grow on a wide range of nitrogen sources, with respect to laboratory strains. Through phenotype microarray analysis, Bou Zeidan et al. (2014) showed that flor yeast can metabolize a wide range of nitrogen sources, including different dipeptides. The presence of *FOT* genes that code for oligopeptide transporters and were acquired by horizontal transfer from *Torulaspora microellipsoides* in wine strains, confers the ability to better use the nitrogen resource of grape must, which results in a competitive advantage (Damon et al., 2011; Marsit and Dequin, 2015; Marsit et al., 2016). As *FOT* genes have been shown for several flor strains (Marsit and Dequin, 2015), their presence might favor the adaptation of these strains to the nitrogen-limited environment during flor aging.

**FIGURE 3:**
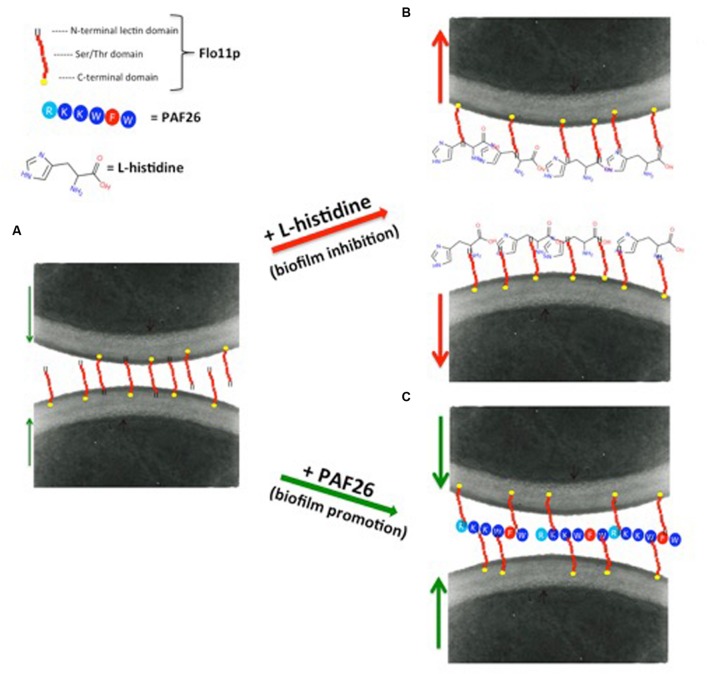
**Proposed models for promotion and inhibition of biofilm formation by flor yeast. (A)** The large and highly glycosylated extracellular N-terminal domain of Flo11p confers high hydrophobicity and negative net charge to the yeast cell wall, and is responsible for cell-to-cell adhesion and biofilm formation. **(B)** Biofilm inhibition. Among the 20 naturally occurring amino acids, L-histidine is a cationic amino acid with a unique imidazole ring as a side chain. This feature of L-histidine might induce the loss of cell adhesion and biofilm formation of the flor strains, by providing non-specific physical interactions with the embedded cell-wall components in general, and with the highly *O*-mannosylated cell-wall mannoprotein Flo11p in particular. This would lead to the failure of air–liquid biofilm formation and cell adhesion. **(C)** Biofilm promotion. PAF26 is a highly hydrophobic and cationic peptide. Due to its properties, electrostatic and hydrophobic interactions can be established between PAF26 and Flo11p. Following this hypothesis, PAF26 would act by facilitating and bridging the Flo11p-mediated interactions between cells, and thus increasing biofilm formation. Red arrows indicate cell to cell repulsion; green arrows indicate cell to cell attraction.

Remarkably, Bou Zeidan et al. (2014) observed that flor strains cannot metabolize dipeptides containing L-histidine, and showed a novel role of L-histidine in the dramatic reduction of biofilm formation and adhesion to polystyrene. Dose-response analysis in nutrient-rich medium showed that L-histidine reduces growth rates, delays the lag-phase, and finally inhibits the growth of the strains tested. Other studies have reported that L-carnosine, which is an L-histidine-containing dipeptide with potential antineoplastic effects (Letzien et al., 2014), can slow down cell growth rates and can kill yeast cells in fermentative metabolism (Cartwright et al., 2012). Interestingly, according to Letzien et al. (2014), L-histidine mimics the effects of L-carnosine, although it shows a stronger effect. Contrary to what was observed in glucose-rich medium, in ethanol medium, the presence of 10 mM L-histidine was sufficient to completely inhibit biofilm formation and adhesion to polystyrene, although these major inhibitory effects were not accompanied by any reduction in cell viability. Moreover, they did not correlate with the transcription level of *FLO11*, which was stable in the absence or presence of L-histidine. L-histidine is a cationic amino acid, with a unique imidazole ring as a side chain that shows high affinity for cationic metals, aromatic amino acids, and many other compounds (Shimba et al., 2003; Liao et al., 2013). By promoting non-specific physical interactions with embedded cell-wall components in general, and with the highly O-mannosylated cell-wall mannoprotein Flo11p in particular, these features might induce the loss of cell adhesion and the failure of air–liquid biofilm formation (**Figures 3A,B**).

Bou Zeidan et al. (2013) showed that a small peptide, PAF26, can promote biofilm formation. PAF26 is a short cationic and tryptophan-rich peptide with cell-penetrating and antifungal activities. It interacts with flor wine yeast without substantial cell death, and also promotes biofilm formation, thus indicating that the peptide interactions and cell death are not necessarily linked. The increased formation of biofilm in the presence of PAF26, and the absence of biofilm formation in the PAF26-treated Δ*flo*11 mutant, indicate that PAF26 requires the presence of Flo11p (**Figure 3C**). Flo11p is the main molecular target for PAF26 in ethanol-rich medium, but not in glucose-rich medium, possibly because with glucose-rich medium, the *FLO11* gene is induced solely during the stationary phase, when the cell concentration is high, and after PAF26 has completed its actions (Swinnen et al., 2006). Bou Zeidan and co-workers also observed that the enhancement of biofilm by PAF26 is independent of *FLO11* gene regulation, but requires expression of a functional *FLO11* gene. Therefore, the effects of PAF26 on biofilm is related to the enhancing of cell-to-cell aggregation by PAF26 under specific biofilm-forming conditions. Similar data were obtained in *C. albicans*, where the peptide LL-37 results in cell aggregation and prevention of cell adhesion (Ibeas et al., 2000).

## Biotechnological Applications of Flor Yeast

The potential applications of flor yeast in wine and other industries might be widened by their immobilization in rigid pla-tforms. Yeast immobilization provides a wide range of advantages compared to the use of free yeast; e.g., yield improvements, feasibility of continuous fermentation processing, and yeast reuse (Kourkoutasa et al., 2004).

**FIGURE 4:**
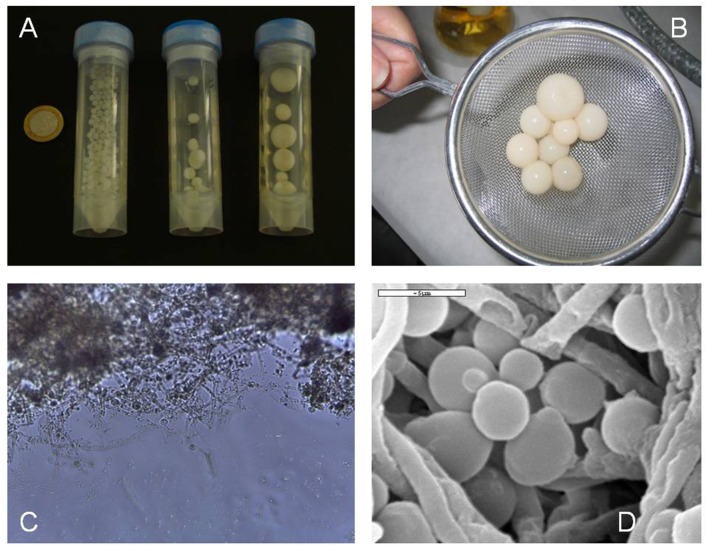
**Biocapsules of flor yeast. (A)** Biocapsules of variable sizes obtained using an orbital shaker at 250, 200, and 150 rpm (left to right) for 7 days. **(B)** Biocapsules removed with a sterile strainer. **(C)** Biocapsule image under an optical microscope at 40× magnification. **(D)** Scanning electron micrograph showing immobilized yeast cells entrapped in the hyphae of the filamentous fungus.

Novel possibilities for the exploitation of flor yeast in other fermentative processes based on spontaneous immobilization within a fungal hyphae framework (*Penicillium chrysogenum*) have been recently attempted (Peinado et al., 2006). The higher immobilization efficiency of flor yeast *versus* non-flor yeast on filamentous fungi has been well demonstrated. Co-immobilization was carried out in a medium containing gluconic acid (as the carbon source for *P. chrysogenum*, and not for flor yeast) in the absence of physico-chemical external support or chemical binders. The immobilization bodies thus obtained (i.e., yeast biocapsules) are hollow, smooth, elastic, strong, creamy-colored spheres of variable sizes, depending on the particular shaking rate and time in the co-immobilization medium (García-Martínez et al., 2011) (**Figure 4**). The biocapsule wall consists of yeast cells bound to fungal hyphae that are trapped. When biocapsules are placed in a medium containing fermentable sugars, the yeast cells colonize and invade all of the hyphae, thereby causing the fungus to die and thence to remain as a mere inert support for the yeast, which facilitates the subsequent reuse of the biocapsules.

Yeast-cell immobilization on *P. chrysogenum* and the suitability of the immobilized biocatalysts for sweet wine production was confirmed by the satisfactory operational stability during repeated batch fermentations of must of dried grapes (García-Martínez et al., 2015). The wines obtained by the fermentation of raisin musts contained greater amounts of volatile compounds. Successive reuse of the immobilized flor yeast revealed a gradual adaptation to the fermentation conditions and an increasingly uniform behavior, in terms of the fermentation kinetics and production of metabolites. Immobilized yeast cells produced higher concentrations of carbonyl compounds, esters and polyols than free yeast cells, and the opposite was true for higher alcohols. The nitrogen compounds (e.g., free amino acids, total aminic nitrogen, ammonium ions, urea) depended on the state of the cells (i.e., free or immobilized), and also on the number of times the yeast had been used.

Flor yeast immobilization might provide some advantages toward obtaining the desired ethanol levels by the easier removal of the yeast cells from the medium, or by reductions in the production costs in the inoculum preparation. Recently, different fungus–yeast combinations have also been attempted by using the Zygomycetes (i.e., *Rhizopus* sp.) (Nyman et al., 2013) as well as using non-flor forming yeast strains for different biotechnological purposes, such as for sparkling wine and sweet wine production (López de Lerma et al., 2012; García-Martínez et al., 2013; Puig-Pujol et al., 2013). The operational stability of the immobilization system proposed might enable its use at a commercial scale for the production of sweet wine (García-Martínez et al., 2015).

## Conclusion

Based on a survey of the most recent literature, flor yeast have emerged as a promising biological model for the study of yeast speciation and phylogenesis, of alternative life-styles in the microbial world, of management of microbial biofilms, and of biofilm industrial applications. The use of microsatellite genotyping has revealed that flor yeast are a group of *S. cerevisiae* strains close to wine strains, and given the contrasting life-styles of these two groups, this makes for an interesting model for the study of yeast adaptation to anthropic niches. Comparative genome hybridization only revealed two genes amplified in the genome of flor strains, which implies that other sources of allelic variations, such as single nucleotide polymorphisms, might explain the specific properties of flor strains and should be explored through population genomics strategies.

The exploitation of other comparative -*omic* tools has provided novel knowledge on several features of flor yeast, and has revealed that proteome remodeling under biofilm-forming conditions might also be related to the production of aroma-properties-related metabolites. Transcriptomic analysis associated to genetic quantitative analysis might deepen this knowledge, and also help to decipher the complex regulatory networks associated with flor aging. The use of flor yeast as a biological model for the study of the management of biofilms is very promising considering that the control of biofilm formation through the use of small molecules is of great interest not only in the biomedical field, but also for practical applications in industrial settings. For example, by using plastic coatings that release small inhibitory molecules, it might be possible to prevent biofilm formation. On the contrary, the use of small molecules that promote biofilm formation can be beneficial to enhance the biological maturation and aging of different foods and beverages. The use of bio-immobilization systems will certainly widen the spectrum of possible applications of flor yeast, which will open new perspectives for fermentation processes, with substantial technical and economic advantages over traditional fermentation methods based on free yeast cells (García-Martínez et al., 2012). Future insights into the role of the *FLO11* gene in flor yeast will also help to improve cell-immobilization technologies (Nedovic et al., 2015).

## Author Contributions

JMG, TG-M, JCM, JM wrote “Specificities of flor yeast: further insights from proteomic and metabolomics” and “Biotechnological applications of flor yeast”; J-LL, SD wrote”Genetic diversity indicates that most flor yeast share the same origin” and “Adaptation of flor yeast and copy-number variations”; SZ, GZ, IM, ALC, MBZ, MB wrote “Introduction”, “Flor yeast as a biological model for the study of small molecules that inhibit or promote biofilm formation” and “Conclusion”. MB coordinated the work and all authors critically revised the manuscript before submission.

## Conflict of Interest Statement

The authors declare that the research was conducted in the absence of any commercial or financial relationships that could be construed as a potential conflict of interest.
